# Rapid commercial CTX-M diagnostics: Performance, limitations and clinical impact

**DOI:** 10.1007/s10096-025-05333-z

**Published:** 2025-11-04

**Authors:** Claudia Aldeia, Gisele Peirano, Johann DD Pitout, Andrea Endimiani

**Affiliations:** 1 Institute for Infectious Diseases (IFIK), University of Bern, Friedbühlstrasse 25, CH-3001, Bern, Switzerland; 2 Division of Microbiology, Alberta Precision Laboratories, Calgary, AB Canada; 3 Department of Pathology and Laboratory Medicine, Cummings School of Medicine, University of Calgary, Calgary, AB Canada; 4 Department of Medical Microbiology, University of Pretoria, Pretoria, Gauteng, South Africa

**Keywords:** CTX-M, ESBL, Diagnostic, Rapid, Commercial, Outcome, Detection, Bloodstream

## Abstract

CTX-M enzymes account for more than 90% of all extended-spectrum β-lactamases (ESBLs) identified in Enterobacterales. Therefore, rapid identification of these enzymes could improve clinical outcomes in patients infected or colonized by such pathogens. In this review, we described the characteristics and limitations of commercially available rapid tests for detecting CTX-M proteins (lateral flow immunoassays) or *bla*_CTX−M_ genes (microarrays, quantitative PCR, or loop-mediated isothermal amplification). Additionally, we summarized and discussed their potential clinical impact. Some commercial CTX-M assays - particularly those analyzing aliquots from positive blood cultures (i.e., Verigene, BioFire FilmArray, ePlex) - demonstrated clear advantages over standard-of-care methods, shortening the interval to effective therapy and improving overall patient outcomes. However, the widespread adoption of these rapid assays in routine laboratories remains limited due to several factors, including high costs and the lack of robust evidence supporting their positive impact. To address these implementation challenges, laboratories should focus on a defined patient subgroup in whom the application of these assays is likely to yield the greatest clinical impact. In particular, we propose that all laboratories at least perform rapid CTX-M assays on all Gram-negative-positive blood cultures (including those with sterile fluids) obtained from critically ill patients, such as ICU-patients with septic shock. This strategy is best when accompanied by active communication between the laboratory and key stakeholders in patient management. Providing rapid results for this subpopulation of patients may facilitate timely initiation of appropriate therapy and ultimately improve patient outcomes.

## CTX-M β-Lactamases: An overview

A cefotaxime-resistant and ceftazidime-sensitive *Escherichia coli* isolate was obtained from the ear exudate of a 4-month German child during 1989 that contained a non-TEM and non-SHV β-lactamase inhibited by clavulanic acid. This enzyme was named CTX-M which stands for active on **C**efo**T**a**X**ime, first isolated in **M**unich [1, 2].

CTX-Ms are extended-spectrum β-lactamases (*ESBLs*) belonging to Amber class A and include more than 280 different enzymes divided into 5 clusters based on their amino acid identities (clusters CTX-M-1, −2, −8, −9 and − 25) [3]. Certain CTX-M enzymes (especially CTX-M-1, CTX-M-9, CTX-M-14) are more active against cefotaxime and ceftriaxone than against ceftazidime and cefepime [4]. Point mutations around the active site of CTX-M-15 and CTX-M-27 have significantly increased their ability to hydrolyze ceftazidime [5]. Notably, CTX-M enzymes originate from *bla*_KLU_-like genes that are present on the chromosomes of different *Kluyvera* spp. [6].

During the 1990’s, *bla*_CTX-Ms_ were reported in various members of Gram-negative (*GN*) bacteria (e.g., *Klebsiella pneumoniae*, *Salmonella* spp.) from South America and Europe [4]. In early 2000, *E. coli* producing CTX-M enzymes have emerged as important causes of global community-onset infections, especially with the urinary tract as a source [7]. Reports from the mid to late 2010 s showed that CTX-M-producing Enterobacterales (mainly *E. coli* with *bla*_CTX-M-15_) have high frequencies (more than 50% of all *E. coli* isolates) in developing countries [8]. It is likely that limited access to basic sanitary facilities combined with human migration had contributed to the spread and high prevalence of *E. coli* with *bla*_CTX-Ms_ in several developing countries [9]. Currently, the most widely distributed with the highest global frequencies among Enterobacterales are CTX-M-15, CTX-M-14 and CTX-M-27 [8, 10]. Enterobacterales with *bla*_CTX-M_ can also contain carbapenemases (e.g., *bla*_KPCs_, *bla*_OXA-48)_, but the over frequency is less than 10% [11].

Mobile genetic elements have played key roles in the capture and dispersal of CTX-M-encoding genes [12]. The insertion element IS*Ecp1* was responsible for the initial capture and mobilization of *bla*_KLU_-like genes from the chromosomes of *Kluyvera* spp. onto plasmids; IS*Ecp1* also served as a strong promoter for the expression of *bla*_CTX-Ms_ [13]. With the aid of another insertion element, namely IS*26*, class 1 integrons or IS*CR1*, IS*Ecp1* have mobilized CTX-M-encoding genes onto different types of plasmids, mainly IncF types as well as IncK, IncI1, IncN, and IncHI2 [6, 12].

Several multidrug-resistant (*MDR*) *E. coli* high-risk clones (e.g., ST10, ST38, ST315, ST393, ST405 and ST648) were responsible for the global dissemination of CTX-M genes [12]. However, ST131 was pivotal in the global emergence, spread and explosive increase of CTX-Ms during the mid-2000s and early 2010s [14].

## Commercial CTX-M diagnostics

Recognition of ESBL-producing Enterobacterales (including those expressing CTX-Ms) has traditionally relied on phenotypic approaches based on minimum inhibitory concentration (*MIC*) determination by microdilution/agar dilution or disk diffusion methods. In particular, culture strains are first tested with conventional antimicrobial susceptibility tests (*ASTs*) and subsequently interpreted according to the criteria of various organizations such as the European Committee on Antimicrobial Susceptibility Testing (*EUCAST*) or the Clinical Laboratory Standards Institute (*CLSI*) [15–17]. Then, using defined AST screening cutoffs set for ESBL detection (e.g., MICs for cefotaxime and ceftazidime > 1 µg/mL according to EUCAST) [18], suspicious strains can be evaluated with further phenotypic confirmatory tests that implement clavulanic acid (e.g., combination-disk test or double-disk synergy test) [19]. Overall, this process is time consuming and the time to results (*TTRs*) from bacterial colonies is usually ≥ 48 h.

To address this issue, many semi-automated and more rapid commercial AST systems are currently available for testing in culture strain [e.g., VITEK 2 (bioMérieux) or Phoenix (Becton-Dickinson) systems with TTRs of ~ 8 h] [20, 21]. Rapid colorimetric [e.g., Rapid ESBL NP test (Liofilchem) with a TTR of < 1 h] and biochemical [e.g., β-LACTA test (Bio-Rad) with a TTR of 2 h and 15 min) assays for ESBL detection are also available [20, 22, 23]. More recently, other automated and rapid AST systems for direct implementation on positive blood culture (*BC*) samples have appeared on the market, such as the Accelerate Pheno (Accelerate Diagnostics) with a TTR of < 7 h [24–26], or the LifeScale (Affinity Biosensors) with a TTR of < 5 h [27]. Nevertheless, none of the above phenotypic-based assays can distinguish CTX-M producers from those producing a different family of ESBLs (e.g., TEM-, SHV-, VEB-, or PER-types). Such distinction can only be accomplished by implementing methods that specifically target the CTX-M proteins or the encoding *bla*_CTX−M_ genes.

In this section, we present non-phenotypic diagnostic systems capable of rapidly detecting CTX-M proteins or *bla*_CTX−M_ genes. In particular, only those that are commercially available and for which a sufficient number of published studies describing their analytical performance to detect CTX-M and clinical impact were included. Notably, although CTX-M ESBLs may be co-produced with carbapenemases, the detection of the latter will not be addressed in detail in this review, as this topic has been recently covered by Simner et al. [28].

### Lateral flow immunoassays (LFIAs)

Antigen-based immunochromatographic tests (*ICTs*) enable detection of enzymes or cell components of bacteria that are associated with antimicrobial resistance [29]. ICTs are often implemented as LFIAs where antigen(s) recognition in a liquid sample is assured by a monoclonal antibody (*mAb*) specific to the target analyte labelled with a visual tag. Results are simply interpreted by visualization of a colored band in the test pad by naked eye. These LFIAs are highly advantageous due to their short TTR (< 30 min), low cost, accuracy, absence of additional instrumentation, ease of use, and minimal hands-on time that make them a paradigm in point-of-care testing [29, 30]. Another key advantage of these rapid diagnostic tests (*RDT*) is their ability to detect proteins (e.g., CTX-M ESBLs), thereby providing evidence of resistance gene expression, whereas gene-based molecular tests (such as those discussed below) do not assess protein expression directly.

#### Performance

In 2020, Bernabeu et al. developed the commercially available NG-Test CTX-M MULTI (NG-Biotech Laboratoires), a LFIA that uses a cocktail of mAbs to detect CTX-Ms of group 1, 2, 8, 9, and 25 in < 15 min (Table 1). This assay was evaluated against a panel of well-characterized Enterobacterales grown on different agar media as well as in positive BCs with GN bacilli. As a result, isolates producing CTX-Ms of the 5 groups were correctly detected from either colonies or directly from positive BCs with both 100% sensitivity (*Se*) and specificity (*Sp*) [32]. The excellent analytical performance of the NG-Test CTX-M MULTI assay and its new version (V2) in detecting CTX-M-producing Enterobacterales (*CTX-M-Ent*) directly from true or spiked positive BCs was later confirmed in several studies [33–35]. This LFIA was further assessed for the rapid detection of CTX-M-Ent directly in urine samples achieving overall Se and Sp of 100% and 99.1%, respectively [36]. Additional commercial LFIAs intended solely for the detection of CTX-Ms are available (RESIST CTX-M, Coris BioConcept; TRURAPID RESIST CTX-M, 3B BlackBio Dx Ltd.). However, no data regarding their performance has been published.

**Table 1:** Characteristics of the commercially available non-phenotypic diagnostic platforms able to rapidly detect *bla*_CTX-Ms_. Only systems with published data were included

System and specific kit (company)	Main technology (method)	Samples tested	Gram stain dependent ^a^	Target organisms	Se/PPA and Sp/NPA for GNs (ranges) ^b^	Antimicrobial resistance targets	Se/PPA and Sp/NPA for bla_CTX−Ms_ (ranges) ^b^	TTR/TAT ^d^	Cost	Clinical impact if CTX-M producers detected
NG-Test CTX-M MULTI (NG-Biotech Laboratoires)	LFIA	Colonies, BCs, Urine	Yes	None	NA	CTX-M groups 1, 2, 8, 9, 25	87.5–100% and 99–100%	<15 min/<2 h	Low	Possible more adequate antibiotic therapy for BSIs
Check-MDR CT103XL (Check-Points Health)	Microarray	Colonies, BCs ^**c**^	Yes	None	NA	*bla*_CTX−M−1/−2/−8/−25/−9_ groups, 6 *bla*_pAmpCs_, 11 *bla*_carbapenemases_, *mcr-1/−2*	98.2–100% and 100%	6–8 h/NA	Medium	Not evaluated
Verigene BC-GN (Diasorin)	Automated gold-nanoparticle microarray	BCs	Yes	9 GNs	94.4–99.4% and 98.3–99.5%	*bla*_KPCs_, *bla*_NDMs_, *bla*_VIMs_, *bla*_IMPs_, *bla*_OXAs_, *bla*_CTX−Ms_	98.9–100% and 100%	< 2.5 h/2.5 h ^**e**^	High	Decreases time to optimal antibiotics for BSIs
Check-Direct ESBL screen (Check-Points Health)	Automated multiplex qPCR	Rectal/peri-rectal swabs	No	None	NA	*bla*_CTX−M_ groups 1, 2, 9, *bla*_SHV−ESBLs_	58.3–95.2% and 92.0–97.6%	< 3 h/NA	Medium	Not evaluated
Acuitas AMR Gene (OpGen)	Multiplex qPCR	Colonies, Urine	Yes	None	NA	47 ARGs including *bla*_CTX−M−1/−2/−9_, *bla*_SHV/TEM_, *bla*_pAmpCs_, *bla*_carbapenemases_, *mcr-1*, *armA*, *sul1/2*, *vanA*	96.7–100% and 99–99.9%	~ 2.5 h/NA	Medium	Not evaluated
Unyvero BCU (Curetis)	Automated multiplex qPCR	BCs	No	11 GPs, 14 GNs, 8 fungi, 1 generic pan target, *Mycobacterium* spp.	100% and 100% (Enterobacterales)	*bla*_CTX−Ms_	74% and 100%	~ 4 h/ 13 h ^**f**^	High	Not evaluated
Unyvero HPN (Curetis)	Automated multiplex qPCR	LRT samples	No	2 GPs, 16 GNs, 2 atypical, *P. jirovecii*	50–100% and 98.4–100% (*K. pneumoniae*)	*bla*_CTX−Ms_	72.7% and NA	~ 4 h/4.6 h	High	Not evaluated
BioFire BCID2 (bioMérieux)	Automated multiplex qPCR and HRM analysis	BCs	No	11 GPs, 15 GNs, 7 *Candida* spp.	98.2% and 98.3% (Enterobacterales)	*mecA/C*, *vanA/B*, *mcr-1*, *bla*_KPCs,_ *bla*_NDMs,_ *bla*_IMPs,_ *bla*_VIMs,_ *bla*_OXA−48−like,_ *bla*_CTX−Ms_	81.8–97.9% and 99.4–100%	~ 1 h/ 3.5–5 h ^**f**^	High	More adequate antibiotic therapy for BSIs
BioFire PN*plus* (bioMérieux)	Automated multiplex qPCR and HRM analysis	LRT samples	No	11 GNs, 4 GPs, 3 atypical, 9 viruses	73–100% and 56.3–99% (*K. pneumoniae*)	*mecA/C* and MREJ, *bla*_KPCs,_ *bla*_NDMs,_ *bla*_IMPs,_ *bla*_VIMs,_ *bla*_OXA−48−like,_ *bla*_CTX−Ms_	Cumulative: 93.8% and 98.4%	~ 1 h/ 2.6 h	High	Possible more adequate antibiotic therapy for LRTs
ePlex BCID-GN (Roche Diagnostics)	Automated gold electrode microarray	BCs, body fluids	Yes	21 GNs, 2 pan targets (GPs and *Candida* spp.)	93.6–100% and 100%	*bla*_KPCs,_ *bla*_NDMs,_ *bla*_IMPs,_ *bla*_VIMs,_ *bla*_OXA−23,_ *bla*_OXA−48,_ *bla*_CTX−Ms_	93.1–100% and 100%	< 2 h/4.6 h ^**e**^	High	Decreases time to optimal antibiotics for BSIs Shorter treatments for BSIs
eazyplex SuperBug CRE (Amplex Diagnostics)	LAMP	Colonies, urine, BCs	Yes	None	NA	*bla*_KPCs,_ *bla*_NDMs,_ *bla*_VIMs,_ *bla*_OXA−48,_ *bla*_OXA−181,_ *bla*_CTX−M_ groups 1/9	100% and 97.9–100%	< 20 min/NA	Medium	Possible more adequate antibiotic therapy for BSIs
eazyplex BloodScreen GN (Amplex Diagnostics)	LAMP	BCs	Yes	4 GNs	95.7–100% and 99–100%	*bla*_CTX−M_ groups 1/9	100% and 100%	< 24 min/NA	Medium	Not evaluated

*LFIA* lateral flow immune assay, *HRM* high-resolution melting, *LAMP* loop-mediated isothermal amplification, *BC* blood culture, *BSIs* bloodstream infections, *LRTs* lower respiratory tract infections, *GN* Gram-negative, *GP* Gram-positive, *Se* sensitivity, *Sp* specificity, *PPA* positive percentage agreement, *NPA* negative percentage agreement, *TTR* time to results, *TAT* turnaround time, *NA* not applicable or not available

^**a**^ If clinical samples directly tested

^**b**^ The standard of care methods (SOCm) used for comparison are imperfect tests. Thus, Se and PPA were taken together, as well as Sp and negative percentage agreement (NPA) [31]

^**c**^ Only the old Check KPC/ESBL was tested for direct use in BCs

^**d**^ The TTR refers to the execution time of the test, whereas the TAT refers to the reporting time to clinicians

^**e**^ After Gram stain (data from clinical studies)

^**f**^ From positive BC (data from clinical studies)

Standard LFIA strips are not adapted for direct use in clinical samples, as the matrix composition can significantly impact Se [29]. In this context, Volland et al. developed the CTX-M BL-DetecTool, an easy-to-use sample preparation device named *SPID* (Sampling, Processing, Incubation, Detection) integrated with the NG-Test CTX-M MULTI strip [37]. This test was validated in a multicenter study across 3 sites for positive BC, urine and pre-enriched rectal swab samples showing Se and Sp of 98.0% and 99.0%, 100% and 100%, 93.3% and 99.3%, respectively. For the same samples, positive (*PPV*) and negative predictive values (*NPV*) were 91.9% and 99.8%, 100% and 100%, 93.4% and 99.3%, respectively. Overall, detection of CTX-Ms was obtained in < 30 min for all biological samples [37]. In another study involving 9 European centers, the CTX-M BL-DetecTool showed consistent analytical performances when tested for the same 3 types of clinical samples [38]. Recently, Nurjadi et al. compared the SPID device (filtration of 1 mL) and simple centrifugation (2 mL) for the pre-treatment of urine samples before using the NG-Test CTX-M MULTI strip. The results of both pre-treatment methods were identical (87.5% Se, 100% Sp), indicating that the use of SPID is suitable for resource-limited areas lacking electricity [39].

#### Limitations

No bacterial species identification (*ID*) is provided by commercial LFIAs. Another limitation of these RDTs is that they do not differentiate between different variants/subgroups of CTX-Ms to which they belong. This aspect is clinically relevant because different CTX-M enzymes hydrolyze β-lactams to varying degrees. For instance, CTX-M-1 exhibits poor hydrolytic activity against ceftazidime, whereas CTX-M-15 efficiently hydrolyzes this antibiotic [5].

Moreover, any new CTX-M variant with an epitope unrecognized by the mAbs subjected to the assay may also result in a false-negative (*FN*) result. Finally, commercial LFIAs detect only CTX-Ms, missing rarer, yet clinically important, ESBLs (e.g., TEM-, SHV-, PER-types) or plasmid-mediated AmpCs (*pAmpCs*; e.g., CMY-types) [29, 40]. For these reasons, the NG-Test CTX-M MULTI was recently combined with the LFIA-CTX-M, an assay using anti-cefotaxime mAb to detect cefotaximase hydrolytic activity after 30 min incubation. The combined LFIA strip (named LFIA Rapid ESC) was evaluated against a well-characterized collection of agar-grown GNs showing 100% Se and Sp for detecting CTX-Ms and other extended-spectrum cephalosporinases [41]. Notably, the LFIA-CTX-M alone had previously shown comparable performance with the commercial Rapid ESBL NP and β-LACTA hydrolysis assays when tested against GNs [42].

One of the major issues of the LFIAs is the false-positive (*FP*) detection (cross-reaction) of target antigens. For instance, *Kluyvera* spp. produce low levels of CTX-M-like enzymes (encoded by *bla*_KLU_ genes) that are the ancestors of the clinically detected CTX-Ms [6]. Therefore, implementation of the LFIAs targeting CTX-Ms may generate FP results [32]. More importantly, the NG-Test CTX-M MULTI showed a high rate of FP results when tested against *Klebsiella oxytoca* complex strains. These species produce the chromosomally encoded OXY β-lactamases that possess amino acid homologies of ∼88% with CTX-Ms, resulting in a cross-reaction with the mAbs included in the LFIAs [43, 44]. Similarly, FP results may also be observed with other species such as *Serratia fonticola*, *Klebsiella aerogenes*, *Citrobacter farmerii* and *Citrobacter amalonaticus* producing FONA, SFO-1, SED-1, and CdiA β-lactamases, respectively [44, 45].

#### Clinical impact

The clinical impact of implementing LFIAs designed to detect CTX-Ms has so far been scarcely evaluated. With the aim to reduce the turnaround time (*TAT*; i.e., time to results reported to clinicians), several authors have applied such LFIAs for the rapid testing of positive BCs coupled with species ID by direct matrix-assisted laser desorption ionization time-of-flight mass spectrometry (*MALDI-TOF MS*) [46].

In an Italian study focusing on bloodstream infections (*BSIs*), the NG-Test CTX-M MULTI was used for positive BCs with *E. coli*. As a result, direct detection of CTX-Ms allowed antibiotic therapy optimization (mainly toward the use of carbapenems), although in 20% of the cases antibiotic escalation was initiated only after receiving the standard AST results. Moreover, CTX-M-negative results prompted antibiotic de-escalations in merely 13.6% of cases. Overall, this work indicated a lack of confidence among treating physicians in RDT results [47]. In another study, direct MALDI-TOF MS and NG-Test CTX-M MULTI were implemented for the rapid diagnosis of urinary tract infections (*UTIs*) due to GNs, with a reporting time to clinicians < 2 h. This approach led to a reduced need of outpatient care with only a marginal increase in costs [48].

### Check-points microarrays

Microarrays offer extensive diagnostic potential given that they can simultaneously detect a large number of target genes and distinguish between their allelic variants through specific hybridization with custom probes [49, 50]. In the past, numerous in-house assays have been designed to characterize antimicrobial resistance genes (*ARGs*), including *bla*_CTX−Ms_ (e.g., [51, 52]). Nowadays, commercial microarray systems have become available. These standardized platforms are easy to operate and can be readily updated.

#### Performance

The Check-Points Health company has extensively developed DNA microarray platforms for the detection of major *bla* genes. Since 2010, several kits testing the presence of *bla*_CTX−Ms_ were released (i.e., Check KPC/ESBL; Check-MDR CT101, CT102, and CT103XL). In particular, the first-generation assays were capable of distinguishing various *bla*_CTX−M_ groups and variants. Moreover, the arrays detected in parallel additional clinically and epidemiologically relevant ESBL, pAmpC and carbapenemase genes. Overall, excellent Se (96.6%−100%) and Sp (100%) were recorded for *bla*_CTX−Ms_ detection when culture strains were tested. Moreover, *bla*_CTX−Ms_ were usually classified into the correct family groups, while no cross-reaction with the OXY enzymes (produced by *K. oxytoca* group strains) was noted [53–56].

The latest microarray kit introduced by the company (Check-MDR CT103XL) can detect and characterize numerous ESBL (including CTX-M-1 group, CTX-M-1-/-15-/-3-/-32-like, CTX-M-2/-8/-25/-9 groups and TEM- or SHV-types), 6 pAmpC, and 11 carbapenemase (GES, GIM, IMP, KPC, NDM, OXA-23/-24/-48/-58, VIM, SPM) genes, along with the *mcr-1*/*-2* that confer resistance to colistin (Table 1) [57]. When validated with culture strains, the Check-MDR CT103XL demonstrated high overall Se and Sp for most *bla* targets. In particular, 98.2–100% Se and 100% Sp in detecting *bla*_CTX−Ms_ were recorded; all *bla*_CTX−Ms_ were also categorized in the appropriate family group [56, 58].

The earlier Check KPC/ESBL kit was also employed to detect ESBL and KPC genes directly from positive BCs. When DNA was isolated implementing a water lysis approach, combined Se and Sp of 94.4% and 100% were recorded, respectively [59]. In a separate study, Juiz et al. used the CT102 Check-MDR kit to analyze BCs positive for *E. coli* or *K. pneumoniae*, including CTX-M-1/-9 group producers. As a result, the array showed 100% agreement with the PCR/sequencing characterization of the strains [60].

#### Limitations

Although commercial microarrays demonstrate exceptional analytical performances, including partial allelic differentiation, they are expensive, labor-intensive and time consuming (i.e., TTR of 6–8 h). Another drawback is that the probes in Check-Points microarrays designed to detect pAmpCs may cross react with chromosomal AmpCs (*cAmpCs*) present in certain Enterobacterales [54]. Equally important, these kits do not detect the bacterial pathogen associated with the target ARGs.

#### Clinical impact

Due to the above limitations, the impact of testing clinical samples with the above commercial microarrays remains unexplored. In our view, Check-MDR CT103XL remains a reliable tool for the rapid characterization of large collections of agar-grown MDR GNs for epidemiological purposes. On the other hand, automated microarray-based platforms implementing syndromic panels have recently shown their potential in clinical applications (see below).

### Verigene system

The Verigene system (Diasorin) is an automated multiplex gold-nanoparticle microarray-based platform for direct analysis of positive BCs (350 µL). This RDT requires a disposable kit and cartridge, the latter inserted into the Processor *SP* (5 min hands-on time) that carries out the extraction of DNA, amplification, and microarray reactions. Assays for Gram-positive (*GP*) and GN pathogens are available in separate kits that are used following Gram staining for positive BCs. A TTR < 2.5 h is obtained by placing the processed cartridge into a dedicated reader [61, 62].

#### Performance

The Gram-Negative Blood Culture Test (BC-GN) kit can simultaneously identify 7 major Enterobacterales, *Pseudomonas aeruginosa*, *Acinetobacter* spp. and the ARGs *bla*_KPCs_, *bla*_NDMs_, *bla*_VIMs_, *bla*_IMPs_, *bla*_OXAs_ and *bla*_CTX−Ms_ (Table 1).

In a series of evaluation studies, BC-GN showed an agreement with the standard-of-care methods (*SOCm*) of 93.7–99.5% for target organisms (Se and Sp of 94.4–99.4% and 98.3–99.5%, respectively). For the *bla*_CTX−Ms_ detection, the agreement was 98.9–100% (Se and Sp of 98.9–100% and 100%, respectively) [63–69]. Regarding the TAT (after Gram staining), BC-GN results were reported within 2.5 ± 1.3 h compared to the 73.6 ± 40.0 h of the SOCm [70].

#### Limitations

The BC-GN assay is unable to distinguish the *bla*_CTX−M_ family groups. It can also be hypothesized that some new *bla*_CTX−M_ variants, emerging post-kit development, may go undetected [61, 62]. Moreover, as for other molecular tests, BC-GN may misinterpret *Kluyvera* spp. strains as CTX-M-producing *K. oxytoca* [71]. Additionally, although epidemiologically less important than CTX-Ms, other ESBLs (e.g., those of TEM- and SHV-types) or pAmpCs (e.g., CMY-type) are not detected. Finally, the high cost per assay could discourage routine implementation of the system.

#### Clinical impact

Numerous studies have demonstrated that implementing the Verigene BC-GN kit has a significant positive clinical impact on BSIs due to target GNs, leading to shorter length of hospital stay (*LOS*), lower mortality rate, and more rapid effective therapies [61]. Nevertheless, specific information regarding the BSIs due to *bla*_CTX−M_-positive strains can only be extrapolated from several studies that provided stratified data on cases due to ESBL producers (i.e., with *bla*_CTX−Ms_, but not carbapenemase genes).

Walker et al. showed that the time to implement effective therapies for BSIs due to ESBL producers was achieved more rapidly with the BC-GN kit than with SOCm (mean 7.3 vs. 41.4 h, *P* = 0.04). However, LOS and 30-day mortality were not significantly improved (12.0 vs. 13.5 days, *P* = 0.59; 26.7% vs. 0%, *P* = 0.11; respectively) [72]. Consistent results were reported by Mahrous et al. (i.e., time to targeted antibiotics: 14.8 vs. 40 h, *P* = 0.021; LOS: 5 vs. 4 days, *P* = 0.17, respectively) [73]. In a multicenter retrospective cohort study, the application of the BC-GN kit alongside antimicrobial stewardship practices significantly reduced the time to optimal antibiotic therapy compared to SOCm (17.1 vs. 28.8 h, *P* < 0.001) [74].

### Check-direct ESBL screen

Quantitative PCR (*qPCR*) consists of the amplification of target gene(s) coupled with the detection of the exponentially amplified DNA product by various methods, such as fluorescence emission with SYBR Green or TaqMan probes. This approach avoids time consuming steps (e.g., running gels), is sensitive, reliable, cost-effective and well-predisposed for multiplexing approaches. Modern apparatuses can also perform high-resolution melting (*HRM*) curve analyses of the DNA products, providing information on single-nucleotide polymorphism(s) present in the amplicon sequence (e.g., characterization of gene variants) [75, 76]. Many in-house qPCR-based assays for detecting ESBL, pAmpC, carbapenemase, and other ARGs have been developed in the past [15, 77]. However, at present, commercially available platforms offer standardized procedures and kits.

#### Performance

The Check-Direct ESBL screen kit (Check-Points Health) is a multiplex qPCR test that detects CTX-M-1, CTX-M-2, CTX-M-9 groups and SHV-type ESBL (SHV-ESBL) genes directly from peri-/rectal swabs. The TTR is < 3 h, including the genotypic differentiation of the targeted *bla*_CTX−M_ groups based on probes labeled with distinct fluorescent dyes. The kit can be adapted to the BD MAX system (Becton-Dickinson), a diagnostic platform which operates as an open qPCR instrument, allowing automated processing of samples and final interpretation (Table 1).

For rectal swabs, Check-Direct ESBL screen displayed overall Se, Sp, PPV and NPV of 58.3–95.2%, 92.0–97.6%, 40.9–71.4%, and 94.3–99.7%, respectively, when compared to direct selective culture followed by molecular characterization of the strains [78–80]. Although most of the strains involved in these studies were CTX-M producers, detailed data on the detection performance of *bla*_CTX−Ms_ are not available.

#### Limitations

The Check-Direct ESBL does not indicate the species ID associated to the target ARGs. Moreover, for the detection of ARGs it shows an overall low Se. However, this is primarily due to the inability to detect off-panel TEM-type ESBL (TEM-ESBL) or pAmpC genes, whose prevalence may vary by region and testing setting.

Another challenge is the relatively high false detection rate, with 3.1–6.9% of qPCR results classified as FPs (i.e., negative by culture). It is generally considered that this phenomenon mainly arises from residual DNA of non-viable bacteria and/or from the lower sensitivity of selective culture approaches without pre-enrichment compared to qPCR [78–80]. Moreover, it can be hypothesized that FPs may also arise from intestinal bacteria that naturally harbor closely-related *bla*_CTX−M_-like genes (e.g., those harbored by *Kluyvera* spp. or *K. oxytoca* group) [52, 81].

#### Clinical impact

Compared to the direct selective culture [82], the Check-Direct ESBL reduced the TAT for peri-/rectal swab screening from 18 to 24 h to only 3–4 h. However, specific data regarding the clinical impact (e.g., infection control management) after implementation of this commercial qPCR kit is not available.

### Acuitas AMR gene panel

The Acuitas AMR Gene Panel (OpGen) kit is a multiplex qPCR in a 96-well format that detects 47 ARGs involving 9 antibiotic classes such as carbapenems (e.g., *bla*_KPC_, *bla*_NDM_, *bla*_OXA−48_, *bla*_IMP_, *bla*_VIM_), cephalosporins (e.g., *bla*_CTX−M−1/−2/−9_, *bla*_TEM−ESBL/SHV−ESBL_, *bla*_CMY_), aminoglycosides (e.g., *aac*, *aph*, *aad*, *armA*), fluoroquinolones (*gyrA* mutations), sulfonamides (*sul1/2*), glycopeptides (*vanA*), and polymyxins (*mcr-1*).

The kit is used with the QuantStudio 5 (Thermo Fisher) qPCR instrument after performing DNA extraction from a culture strain with the QIAGEN EZ1 Advanced XL system (30 min hands-on time). The Acuitas Lighthouse software enables interpretation of results. In particular, it is able to predict the antibiotic resistance phenotype for *Enterococcus faecalis*, *P. aeruginosa* and major Enterobacterales. Such species should be manually inserted in the system after obtaining their ID through separate SOCm (e.g., MALDI-TOF MS). The total TTR is ~ 2.5 h (Table 1) [83, 84].

#### Performance

The Acuitas AMR Gene Panel was evaluated for the U.S. Food and Drug Administration clearance in a large multicenter study comparing the results with both SOCm and whole-genome sequencing (*WGS*). Overall, positive (*PPA*) and negative percent agreement (*NPA*) for agar-grown strains were ≥ 94.4% and ≥ 96.5%, respectively. In particular, PPA of 96.7–100% and NPA of 99.0–99.9% were observed for the *bla*_CTX−M_ targets [84]. In another investigation, 44 MDR *K. pneumoniae* isolates (of which 28 *bla*_KPC_-positive) were characterized with both Illumina WGS and Acuitas system. The latter produced highly concordant ARG results and clustering analysis, but in significantly less time than WGS (i.e., no need for computational resources and bioinformatic analyses) [83].

The Acuitas AMR Gene Panel was compared to the Check-MDR CT103XL microarray by testing a collection of Enterobacterales possessing various *bla* genes characterized by Illumina WGS. The overall Se and Sp for the generic detection of *bla*_CTX−Ms_ were 100% and 100% for the Acuitas AMR and 98% and 94% for the Check-MDR CT103XL, respectively. While both systems showed 100% Se and Sp for the detection of *bla*_CTX−M−9/−14_, Acuitas outperformed Check-MDR CT103XL in detecting *bla*_CTX−M−1/−15_ (100% Se and 100% Sp vs. 97% and 92%, respectively) [85].

In a multicenter study, the Acuitas AMR Gene Panel was also evaluated directly in urine samples. When the uropathogens were *E. coli*, *K. pneumoniae* or *P. mirabilis*, the overall accuracy, PPA and NPA in predicting antibiotic resistance profiles based on the detected ARGs were 91–93%, 65–88%, and 94–97%, respectively. Of note, for *E. coli* and *K. pneumoniae*, the overall accuracy, PPA and NPA of the Acuitas Lighthouse software in predicting cefotaxime resistance (an indicator of ESBL production) were 94–98%, 78–88%, and 98–100%, respectively [86].

#### Limitations

The Acuitas system is not yet fully automated. It requires approximately 30 min for the preparation of genomic extracts before implementing the qPCR kit.

#### Clinical impact

The potential benefits of using the Acuitas AMR Gene Panel directly on clinical samples (e.g., BCs, urine) have yet to be evaluated.

### Unyvero system

The Unyvero system (Curetis) is a multiplex qPCR-based RDT automated platform consisting of 3 hardware units: lysator for genomic extraction (30 min), analyzer for qPCR analyses, and cockpit to manage testing process (display, store, and transmit results). The overall hands-on time is < 2 min, with a TTR of ~ 4 h. Five specific syndromic cartridges for positive BCs (BCU), hospitalized pneumonia (HPN), intra-abdominal (IAI), implant & tissue (ITI), and urinary tract infections (UTI) are available. Notably, according to the bacterial species and ARGs detected, the Unyvero system indicates a possible resistance phenotype [87].

#### Performance

The Unyvero BCU cartridge detects 11 GPs, 14 GNs (including 9 major Enterobacterales), 8 fungi, *Mycobacterium* spp., and it also includes a universal bacterial target. Furthermore, it identifies the following ARGs: *vanA/B*, *mecA/C*, *aac(6’)/aph(2’’)*, *aacA4*, *ermA*, *bla*_KPCs,_ *bla*_NDMs,_ *bla*_IMPs,_ *bla*_VIMs,_ *bla*_OXA−48−like,_ *bla*_OXA−23_, *bla*_OXA−24/40_, *bla*_OXA−58_ and *bla*_CTX−Ms_ (Table 1).

The BCU cartridge was evaluated in two studies where SOCm were used as reference tests. In a multicenter assessment, 100% Se, Sp, PPV and NPV were recorded for Enterobacterales species detection; all *bla*_CTX−M_-positive strains were also identified. The overall TTR from positive BCs was 13 h and 24 min for the BCU compared to 47 h and 54 min for the SOCm [88]. In another study focusing on polymicrobial BSIs (2 to 5 bacteria), the BCU assay correctly identified all bacteria of the mix in 72.3% of the samples. However, the BCU kit was unable to detect *bla*_CTX−M−14_, *bla*_CTX−M−15_, *bla*_CTX−M−79_, or *bla*_CTX−M−174_ in 7 isolates belonging to Enterobacterales (74.1% Se, 100% Sp) [89].

The Unyvero HPN cartridge identifies 2 GPs, 16 GNs (including 10 major Enterobacterales), 2 atypical bacteria, *Pneumocystis jirovecii* and the following ARGs: *mecA/C*, *ermB*, *sul*1, mutations encoding GyrA83 and GyrA87, *bla*_KPCs,_ *bla*_NDMs,_ *bla*_IMPs,_ *bla*_VIMs,_ *bla*_OXA−48,_ *bla*_OXA−23_, *bla*_OXA−24/40_, *bla*_OXA−58_, generic *bla*_TEM/SHV_ and *bla*_CTX−Ms_ (Table 1). Pathogens are reported semi-quantitatively as +, ++, and +++ [90].

The analytical performance of the HPN cartridge for lower respiratory-tract (*LRT*) samples has been evaluated in numerous studies where the SOCm were used as reference. For all target bacteria, concordant results, Se/PPA and Sp/NPA ranged between 71 and 97.8%, 81–96.5% and 92–99.6%, respectively. The same parameters for *K. pneumoniae* - the major *bla*_CTX−M_ carrier for hospital-acquired pneumonia (*HAP*) – were 75–100%, 50–100%, and 98.4–100%, respectively [81, 87, 91–95]. For the detection of *bla*_CTX−Ms_, Peiffer-Smadja et al. showed that the HPN cartridge identified only 5 out of 8 (63%; 72.7% Se) of the CTX-M-Ent detected with the SOCm; moreover, the time for recognition was 4.6 h [81].

Remarkably, all studies consistently emphasized that the Unyvero HPN assay reported additional potential pathogens compared with the SOCm (e.g., between 10.6% and 25.3% more in [87, 94–96]). Such organisms were classified as FPs, affecting the statistical analyses of analytical performance. However, Klein et al. clearly demonstrated that most (84.9%) of these FP organisms represented true detections when confirmed with PCR/sequencing of the original specimens. A similar issue was observed for *bla*_CTX−Ms_. The Unyvero HPN cartridge identified *i*) *bla*_CTX−Ms_ along with species not detected with the SOCm or *ii*) *bla*_CTX−Ms_ that were carried by species not on the Unyvero panel (e.g., *Providencia stuartii*) [87]. This overall phenomenon can be attributed to the higher Se of Unyvero HPN vs. SOCm, which largely explains the low agreement recorded between the two approaches for both organisms and *bla*_CTX−Ms_ detection. Essentially, SOCm are imperfect standard tests unsuitable for assessing this or other PCR-based RDTs [31, 97].

#### Limitations

An important limitation of the Unyvero assays is their inability to link the detected ARG(s) with the harboring bacterial host. Moreover, detection of *bla*_CTX−Ms_ is problematic: it may fail or, on the other hand, may be classified as FP. Nevertheless, the main issues associated to the Unyvero system are observed with the HPN cartridge. LRT samples, especially sputa, are subject to cross contamination with the upper respiratory flora. This leads to numerous FP outcomes (over-reporting), generating challenging interpretations [97].

#### Clinical impact

The impact of using the Unyvero BCU cartridge for the rapid diagnosis of BSIs has not yet been evaluated. In a randomized study focusing on LRT infections due to GNs, Darie et al. showed that the implementation of the HPN cartridge on bronchoalveolar lavages (*BALs*) significantly reduced the duration of inappropriate antibiotic treatment compared to the SOCm (mean of 47.1 h vs. 85.7 h, respectively; *P* < 0.0001). However, there were no significant differences between Unyvero and SOCm groups regarding clinical stability, hospital discharge, admission to intensive care unit (ICU), or death [98]. Unfortunately, specific data regarding the impact of Unyvero assays on infections due to *bla*_CTX−M_-possessing organisms are not available.

### BioFire filmarray

The BioFire FilmArray (bioMérieux) is a syndromic RDT automated platform that combines DNA extraction from various clinical samples, nested multiplex PCRs and post-qPCR amplicon HRM analyses in a single pouch (only 2 min hands-on time) inserted into the FilmArray TORCH system. The TTR is ~ 1 h, including automated interpretation [62, 99, 100]. Diagnostic kits for gastro-intestinal, meningitis/encephalitis, respiratory, and BSIs are available.

#### Performance

The BioFire Blood Culture Identification (BCID2) kit is approved for direct implementation on positive BCs (0.2 mL) without the need of Gram stain. It simultaneously identifies 43 targets, including 11 GPs, 15 GNs, 7 *Candida* spp. along with *mecA/C*, *vanA/B*, *mcr-1*, *bla*_KPC,_ *bla*_NDM,_ *bla*_IMP,_ *bla*_VIM,_ *bla*_OXA−48−like,_ and *bla*_CTX−M_ ARGs (Table 1). The BCID2 is an optimization of the BCID kit that identified 27 targets, but not *bla*_CTX−Ms_ [62, 100].

In the meta-analysis of Peri et al. (2022), the pooled Se and Sp of the BCID2 for detecting Enterobacterales were 98.2% and 98.3%, respectively [100]. Considering more recent papers, the pooled Se/PPA and Sp/NPA for *bla*_CTX−Ms_ detection were 94.9–97.9% and 99.4–100%, respectively [100–102]. Lately, for BCs positive for *K. pneumoniae* or *E. coli* producing CTX-Ms the assay showed 81.8–90.9% Se and 100% Sp in detecting *bla*_CTX−Ms_ [103].

With regard to the TAT, Sparks et al. showed that the average time from BC collection to species ID with BCID2 was 24.6 h vs. 38.2 h using the MALDI-TOF MS on colonies. Moreover, the TAT for *bla*_CTX−Ms_ was 21.3 h with the BCID2, whereas 50.7 h with the VITEK 2 ASTs followed by in-house PCRs [104]. Two recent studies reported times from positive BCs of 3.5–5 h for BCID2 and 46.4–46.9 h for ID/ASTs obtained with VITEK 2 or MicroScan WalkAway (Beckman Coulter) systems (both *P* < 0.001) [101, 105]. Tatli-Kis et al. stated that the results obtained with BCID2 were on average 1 day, 1 h and 9 min faster than SOCm (*P* < 0.01) [106]. Consistent results were obtained in a more recent study [103].

Claeys et al. compared the performance of the BioFire BCID2 with that of the Verigene BC-GN. Verigene was performed as part of routine clinical analyses, whereas BioFire was tested retrospectively with the same BC samples stored at −80 °C. Compared to the SOCm, Verigene achieved a PPA of 98.8% (81/82 organisms) for the target species, while BioFire had a PPA of 96.2% (94/97 organisms). However, among the 25 organisms absent on the BC-GN Verigene panel, BioFire BCID2 detected 15 (60%) species [107].

The BioFire FilmArray Pneumonia *plus* (PN*plus*) Panel is designed to test sputa or other LRT samples (200 µL). It detects numerous targets: 18 bacteria (11 GNs, 4 GPs and 3 atypical), 9 viruses and the following ARGs: *mecA/C* and MREJ, *bla*_KPCs,_ *bla*_NDMs,_*bla*_IMPs,_ *bla*_VIMs,_ *bla*_OXA−48−like_ and *bla*_CTX−Ms_. Pathogens are reported as 10^4^ to ≥ 10^7^ DNA copies/mL, while ARGs are only listed if the potentially associated organism is detected (Table 1).

In a review including studies performed during 2019–2022, the performance of PN*plus* was compared to the SOCm showing cumulative Se and Sp for the detection of *bla*_CTX−Ms_ of 93.8% and 98.4%, respectively. Of note, 27.6% of the *bla*_CTX−Ms_ detected by PN*plus* were over-diagnosed (i.e., not identified in the isolates found with SOCm) and therefore classified as FP; 6.1% of the *bla*_CTX−Ms_ were also missed. The review also indicated 79–100% Se and 93–100% Sp for the detection of on-panel bacteria [97].

More recent studies with PN*plus* have essentially confirmed its overall analytical performance [108–110]. Several analyses have also shown that Se/PPA, Sp/NPA, and agreement for *K. pneumoniae* detection were 73–100%, 56.3–99% and 20–85.7.7%, respectively [108, 110–113]. Moreover, the total microbiological yield in LRT samples by the PN*plus* was higher compared to SOCm. For instance, 91% vs. 55% (*P* < 0.0001) in sputa or endotracheal aspirate samples [114]; 58.8% vs. 34.8%, (*P* = 0.000) in BALs [108]; 71% vs. 51% (*P* = 0.004) in mixed LRT samples [115]; 95.8% vs. 57.1% (*P* < 0.001) in sputa or BALs, with a mean number of pathogens/samples of 1.99 vs. 1.44, respectively [112]. As for the Unyvero HPN assay, the low agreement recorded between the two approaches is due to the higher Se of PN*plus vs.* SOCm (imperfect standard tests) [31, 97].

In a randomized study, Pool et al. reported TTRs of 1.7 h for PN*plus vs.*. 66.7 h for SOCm (*P* < 0.0001) [115]. Concerning the TAT, a study focusing on community-acquired pneumonia (*CAP*) indicated that it was shorter for the PN*plus* compared with in-house PCR (2.6 h vs. 24.1 h, *P* < 0.001) or SOCm (2.6 h vs. 57.5 h, *P* < 0.001) [114]. In another randomized analysis, the TAT for PN*plus* was 53.8 h shorter than SOCm (*P* < 0.001) [116].

In a multicenter analysis, BioFire PN*plus* Panel and Unyvero HPN cartridge were concomitantly tested and compared to SOCm [90]. The overall positivity rate for both assays exceeded SOCm (74.2% for BioFire and 60.4% for Unyvero vs. 44.2% by SOCm; _*X*_^2^ test *P* < 0.0001), with *E. coli* and *K. pneumoniae* being more frequently detected. Overall, FilmArray showed 89.4–99.3% Se and 93.9–99.9% Sp (98.1% and 97.7% for *K. pneumoniae*, respectively), while for Unyvero 83.9–96.9% Se and > 99% Sp were recorded (88.9% and 99.8% for *K. pneumoniae*, respectively). BioFire detected 14 *bla*_CTX−Ms_ (of which 12 were concordant with SOCm) and failed to detect 3, whereas Unyvero identified 32 *bla*_CTX−Ms_ (of which 17 were concordant with SOCm) [90].

#### Limitations

BioFire BCID2 does not distinguish the *bla*_CTX−M_ groups and is unable to detect further ESBLs (e.g., SHV-/TEM-type ESBLs) or pAmpCs (e.g., CMY-2) [117, 118]. *K. oxytoca* group isolates may be falsely reported as *bla*_CTX−M_-positive [102], while *Kluyvera* spp. can be misidentified as *bla*_CTX−M_-positive *K. oxytoca* [71]. We also emphasize that the high cost of the test may disinvite the use of this platform. However, a study modelling the former BCID kit in comparison with SOCm showed that there could be cost savings and improved quality-adjusted life years in favor of BCID for BSIs due to *E. coli* [119].

In general, the BioFire PN*plus* Panel displays matching intrinsic limitations of the BCID2 kit [97]. In addition, it may not detect *K. aerogenes* [120]. Nevertheless, as emphasized for the Unyvero HPN cartridge, the main issues linked to the use of PN*plus* (i.e., high-frequency of FP results) are generated by the non-sterile characteristics of LRT samples [97].

#### Clinical impact

Most studies evaluating the clinical impact of the BioFire BCID2 showed that its generalized implementation is significantly associated with a lower mean time to effective antibiotic therapy [103, 106, 121–123], hospital LOS [121], and 28/30-day mortality rates [122, 124]. However, only three studies provided specific data regarding the BSI cases due to CTX-M producers.

In a single-centre study, the mean time to effective therapy from BCID2 results was 2.8 h for CTX-M-positive organisms; such time was significantly lower than that recorded during the implementation of SOCm (17.7 h; *P* = 0.0041) [123]. In another monocentric analysis, among patients under non-appropriate empirical treatment for BSIs due to GNs (including ~ 10% due to CTX-M producers), the proportion of transitions to adequate antibiotic therapy after reporting the Gram stain and BCID2 were 50% and 87.5%, respectively (*P* < 0.001). However, the survival rates between the two groups did not reach statistical significance (long-rank test, *P* = 0.10) [125]. A retrospective study indicated that, although a hospital stewardship program was active, only 5 of the 7 BSIs due to CTX-M-producing *E. coli* or *K. pneumoniae* strains were correctly treated (i.e., meropenem) after receiving the BCID2 results. Moreover, for the same two species, but without ARGs, only 61.5% (16/26) of treatments were correct. The authors emphasized that the most frequent mistake was the escalation from ceftriaxone to piperacillin-tazobactam or meropenem therapies [126].

In randomized clinical studies, the generalized (i.e., for all on-panel bacteria) implementation of the BioFire PN*plus* Panel for testing LRT samples showed significantly more adequate antibiotic therapy (including escalations or de-escalations) compared to SOCm. However, there were no differences between the use of PN*plus* and SOCm regarding 30-day and in-hospital mortality, LOS, and readmission within 30 days [115, 116, 127].

Specific data regarding the impact of PN*plus* use for LRT infection exclusively due to GNs are scarce. In a randomized study focusing on GNs, equivalent overall outcomes listed above (i.e., enhanced positive effect on antibiotic therapy) were observed [128]. Moreover, only a prospective/monocentric study have analyzed the impact of PN*plus* for the rapid diagnosis of ventilator-associated pneumonia (*VAP*) due to ESBL producers. As a result, the use of BioFire was significantly associated with optimal empirical antibiotic therapy when compared to SOCm (68% vs. 27%, *P* = 0.001). A mean time of prescription of 9 h for BioFire vs. 30 h for SOCm (*P* = 0.09) was also recorded [129].

### ePlex system

The ePlex (Roche Diagnostics) is an automated RDT system that utilizes 3 specific cartridges for the identification of GP (BCID-GP), GN (BCID-GN) or fungal (BCID-FP) pathogens along with ARGs in positive BCs (TTR < 2 h). Cartridges are selected after performing the Gram stain, loaded with 50 µL of sample (< 1 min hands-on time), and then inserted into the scalable ePlex apparatus. The system performs PCR amplification of target genes that is followed by an exonuclease digestion to obtain single-stranded amplicons, which are then annealed with a ferrocene signal probe. This solution moves inside a microfluidic device within the cartridge where target DNA/probe interact with specific capture probes bound to gold electrodes on a special microarray (eSensor). If hybridization occurs, a voltage signal is detected and automatically interpreted [130].

#### Performance

The ePlex BCID-GN Panel can identify 21 GNs along with KPC, NDM, IMP, VIM, OXA-23, OXA-48 and CTX-M genes; it also generically detects GPs and *Candida* spp. (Table 1). Notably, the original inclusion of the latter two pan-targets adds clinical value as they may identify potential errors in the Gram stain interpretation or presence of potential anaerobic pathogens.

The ePlex BCID-GN has been evaluated in numerous studies where its performance was compared to SOCm. For the detection of target GNs, Se/PPA and Sp/NPA were 93.6–100% and 100%, respectively. For the detection of *bla*_CTX−Ms_, Se/PPA and Sp/NPA were 93.1–100% and 100%, respectively [131–134]. The BCID-GN was also evaluated on positive BC bottles inoculated with body fluids (e.g., CSF, peritoneal, synovial, and pleural fluids). As a result, overall PPA and NPA of 91.7% and 100% were obtained [135].

Data regarding the detection time were provided only in studies combining the 3 ePlex cartridges. Martin et al. indicated that the ePlex was associated with a significantly decreased time to species ID compared to MALDI-TOF MS performed on colonies (23.9 h vs. 45.3 h; *P* < 0.001) [136]. For Caspar et al., the median TTR for ePlex after Gram stain results was 4.6 h (a median of 24.1 h from the sampling of the positive BC). In contrast, the median TTR for species ID and ASTs using SOCm were 28 h and 29.4 h, respectively (both, *P* < 0.001) [137].

Of note, Claeys et al. analyzed the performance of ePlex BCID-GN and Verigene BC-GN using the same positive BCs and comparing the results of both systems to SOCm. Considering the on-panel target species, ePlex and Verigene showed PPA of 98.9% and 98.6%, respectively. However, ePlex was able to identify more GNs than Verigene (i.e., including 55% of those not included in the Verigene panel) [138].

#### Limitations

ePlex BCID-GN presents drawbacks similar to other syndromic platforms (e.g., Verigene and BioFire). It does not distinguish *bla*_CTX−M_ groups and fails to detect SHV-/TEM-type ESBL or pAmpC genes [131]. Moreover, the kit erroneously reported 2 cases of BSI with *Kluyvera* spp. as due to *bla*_CTX−M_-positive *Enterobacter cloacae* complex [71]. Finally, as for the other automated RDT platforms, the elevated cost may disfavor its implementation.

#### Clinical impact

So far, limited studies have focused on this aspect. Moreover, these investigations were performed mixing the results of the 3 ePlex cartridges. In general, there was agreement in stating that the ePlex implementation significantly reduced the time to optimal antimicrobial therapy for BSIs, whereas the effect on costs, LOS and mortality did not seem significantly different when compared to SOCm [136, 137, 139].

In a recent study, Chang et al. compared the pre- (BioFire BCID, not detecting *bla*_CTX−Ms_) and post-implementation of ePlex BCID-GN to retrospectively analyze the outcome of BSIs due to CTX-M-Ent (*n* = 130 vs. *n* = 145, respectively). Accordingly, ePlex showed a significant reduction in the time to appropriate antimicrobial therapy after Gram stain, defined as carbapenem use (44.5 h vs. 7.9 h; *P* < 0.001); a shorter duration of antibiotic treatment was also observed (14.4 vs. 12.7 days, *P* = 0.014). However, LOS (9 vs. 10 days), 30-day readmission (10% vs. 19%) and hospital outcome (e.g., expired: 6.15% vs. 6.9%) did not show statistically significant differences between the two groups [140].

### Eazyplex loop-mediated isothermal amplification (LAMP) system

The LAMP method allows for the amplification and fluorescent detection of target DNA from clinical samples at a constant temperature, eliminating the need for an expensive thermocycler. Remarkably, a typical genomic extraction is not strictly required (simple heat or chemical lysis is sufficient), since this highly sensitive method uses the *Bst* DNA polymerase that is resistant to inhibitors present in serum or heparin [141, 142].

In the past, many authors have designed in-house LAMP assays to detect clinically relevant *bla* genes (e.g., *bla*_NDM_, *bla*_KPC_) [143–145], including the *bla*_CTX−Ms_ belonging to the different groups (e.g., [146–149]). Overall, the LAMP implementation on clinical samples is very rapid (TTR < 1 h), highly sensitive, and with a limit of detection 100–1000 times lower than PCR-based approaches. However, in-house LAMP assays face significant challenges in achieving standardization and routine use in a clinical laboratory context.

#### Performance

The commercially available eazyplex LAMP system (AmplexDiagnostics) comprise a series of freeze-dried and ready-to-use kits coupled with real-time photometric detection of amplified targets using transportable instruments (Genie II, eazyMini, or eazyOne).

The eazyplex SuperBug CRE kit can detect KPC, NDM, VIM, OXA-48, OXA-181 and CTX-M-1/-9 group genes (Table 1). In its first evaluation (2015), this LAMP assay was tested against a well-characterized collection of Enterobacterales: all carbapenemase and/or CTX-M producers were correctly identified within 15 min [150]. The excellent capacity of the SuperBug CRE assay to detect CTX-M-1/9-like producers was further confirmed in additional studies testing agar-grown GNs [151, 152].

The eazyplex SuperBug CRE kit was also directly implemented on urine samples (pellets), of which 60% contained *E. coli* or *K. pneumoniae* strains producing CTX-M-1/-9 group ESBLs. The assay showed Se of 100% and Sp of 97.9%, with TTR < 20 min [153]. These promising results for direct testing of urine samples were partially confirmed by Sekowska et al., who also tested positive BCs with similar outcomes (i.e., 90.9% and 90.0% agreement with standard PCR methods, respectively) [154].

Recently, the eazyplex BloodScreen GN kit (AmplexDiagnostics) has been specifically designed for direct use in positive BCs to detect GNs (*E. coli*, *K. pneumoniae*, *Proteus mirabilis*, *P. aeruginosa*) and *bla*_CTX−M−1/−9_ groups. In a prospective study, this RDT showed 95.7–100% Se and 99–100% Sp for species ID together with 100% Se and 100% Sp for *bla*_CTX−Ms_ detection. The overall TTR was < 24 min [155].

#### Limitations

Only the BloodScreen GN kit provides simultaneous information regarding bacterial species ID. Moreover, both LAMP-based assays do not identify additional ESBL (e.g., those of TEM-/SHV-types) or pAmpC genes and can only discriminate between two CTX-M groups. For the latter issue, Higgins et al. suggested the implementation of the loop-primer endonuclease cleavage LAMP (*LEC-LAMP*) to distinguish *bla*_CTX−M−1_ and *bla*_CTX−M−15_ within the *bla*_CTX−M−1_ group [156].

#### Clinical impact

Although the commercial LAMP-based assays designed to detect CTX-Ms have shown their potential to improve the outcome of infections (i.e., BSIs and UTIs), their clinical impact has been scarcely assessed.

In an Italian study, Fiori et al. showed that the implementation of the eazyplex SuperBug CRE kit coupled with the direct MALDI-TOF MS for species ID on positive BCs significantly improved the outcome of BSIs due to CTX-M-producing *E. coli* and *K. pneumoniae*. In particular, after results were communicated to clinicians (on average 20 h after BC collection), the proportion of appropriate antibiotic treatments increased from 30.0% to 92.0% [157].

## Conclusions

Despite regional variations, CTX-M enzymes account for over 90% of ESBLs in Enterobacterales worldwide [8, 10]. Thus, implementing rapid, user-friendly CTX-M diagnostics could improve clinical outcomes in infected or colonized patients. However, their widespread adoption in routine clinical microbiology laboratories remains limited. Several factors contributed to this restriction, including the high assay costs and insufficient reimbursement. Moreover, the integration into existing laboratory workflows is further constrained by the requirement for additional trained personnel and validation processes. In addition, their clinical utility is sometimes perceived as limited, as robust evidence demonstrating a clear impact on healthcare costs and patients’ morbidity/mortality is still lacking [158, 159].

As a consequence, efforts to promote the adoption of rapid CTX-M diagnostics should prioritize clinical and public health advocacy as well as targeted educational initiatives [158]. The impact of rapid CTX-M detection that improves patient outcomes, and aids with the implementation of effective antimicrobial stewardship, and infection control is currently lacking. Undertaking large, randomized international studies to address these issues, is essential to encourage uptake by clinicians and healthcare institutions. Inclusion of these assays into clinical guidelines and integrating them into surveillance programs could further strengthen their epidemiological and therapeutic relevance. In parallel, educating laboratory personnel and clinicians on the benefits and interpretation of rapid diagnostics can enhance acceptance, support appropriate utilization, and emphasize their role in optimizing antimicrobial therapy while limiting the spread of MDR pathogens.

In light of the present overview, some commercial CTX-M assays - particularly those analyzing aliquots from positive BCs and capable of simultaneously identifying further ARGs (e.g., encoding carbapenemases) along with major bacterial species (i.e., Verigene, BioFire FilmArray, ePlex) - exhibited superior performance compared with SOCm in detecting CTX-Ms along with additional antimicrobial resistance mechanisms. More importantly, by significantly reducing the TAT (Table 1), these tests shortened the interval to effective therapy (especially if an antibiotic stewardship team is in place) and may also lower both LOS and mortality [72–74, 123, 125, 140].

Consequently, it is evident that all patient types could potentially benefit from the integration of rapid CTX-M assays into the laboratory workflow for BCs, alongside the SOCm, which continues to provide valuable information (e.g., full ASTs associated to the species ID). However, to overcome the challenges outlined above, clinical laboratories facing high-prevalence of CTX-M producers should initially target specific patient subgroups in which these assays are likely to achieve the greatest clinical impact. This decision-making process should account for multiple factors (e.g., costs, local epidemiology) and engage all key stakeholders in patient management (e.g., clinicians, antimicrobial stewardship team, pharmacists) [159].

Through a minimalistic implementation strategy, we propose that all clinical laboratories serving tertiary-care hospitals should at least perform rapid CTX-M assays on all GN-positive BCs (including those from sterile fluids) obtained from highly specialized wards caring for critically ill patients (e.g., ICU-patients with septic shock). This strategy is best when paired with an active communication between the laboratory and stakeholders [159]. As shown for Verigene, BioFire FilmArray and ePlex syndromic systems [72–74, 123, 125, 140], providing rapid results in this patient subgroup may facilitate prompt initiation of appropriate therapy and thereby enhancing patient outcomes [159].

## Data Availability

No datasets were generated or analysed during the current study.
